# Inguinal hernia repair in inpatient children: a nationwide analysis of German administrative data

**DOI:** 10.1186/s12893-021-01371-4

**Published:** 2021-10-20

**Authors:** Andreas Heydweiller, Ralf Kurz, Arne Schröder, Christina Oetzmann von Sochaczewski

**Affiliations:** 1 Sektion Kinderchirurgie, Klinik und Poliklinik für Allgemein-, Viszeral-, Thorax- und Gefäßchirurgie, Universitätsklinikum Bonn, Venusberg-Campus 1, 53127 Bonn, Germany; 2 Klinik für Kinder- und Jugendmedizin, Klinikum Dortmund, Dortmund, Germany; 3 Klinik und Poliklinik für Kinderchirurgie, Universitätsmedizin Mainz, Mainz, Germany

**Keywords:** Health services research, Hernia surgery, Inpatients, Hospital reimbursement, Paediatric surgery, Population-based

## Abstract

**Background:**

Contrary to adult inguinal hernia surgery, large-scale investigations using registries or administrative data are missing in paediatric surgery. We aimed to fill this gap by analysing German administrative hospital data to describe the current reality of inpatient hernia surgery in children.

**Methods:**

We analysed aggregated data files bought from the German federals statistics office on hospital reimbursement data separately for principal diagnoses of inguinal hernia in children and for herniotomies in inpatients. Developments over time were assessed via regression and differences between groups with nonparametric comparisons.

**Results:**

Principal diagnoses of hernias were decreasing over time with the exception of male bilateral and female bilateral incarcerated hernias in the first year of life which increased. The vast majority of operations were conducted via the open approach and laparoscopy was increasingly only used for females older than 1 year of age. Recurrent hernia repair was scarce. Rates of inguinal hernia repair were higher in both sexes the younger the patient was, but were also decreasing in all age groups despite a population growth since 2012. The amount of inguinal hernia repairs by paediatric surgeons compared to adult surgeons increased by 1.5% per year.

**Conclusions:**

Our results corroborate previous findings of age and sex distribution. It demonstrates that inpatient hernia repair is primarily open surgery with herniorrhaphy and that recurrences seem to be rare. We observed decreasing rates of hernia repairs over time and as this has been described before in England, future studies should try to elucidate this development.

**Level of evidence:**

III.

## Introduction

Inguinal hernia repair represents the most common operation performed by paediatric surgeons in the United States of America [1]. However, nationwide data have been scarcely presented for paediatric inguinal hernias and were largely restricted to Denmark [2], Taiwan [3], and England [4] based on their large health insurance-based registries. The Swedish registry data has so far only been used to investigate specific narrow research questions in adolescents [5], as has a large insurance database in Japan [6]. Contrary to this situation in paediatric inguinal hernia repair, research in adults may not only rely on large-scale registries from different countries [7], but also on routine administrative data [8] and even the combination of both [9]. Just recently, the epidemiology of inguinal hernia repair in children has been described for the United States of America [10]. We therefore aimed to assess variations over time in paediatric inguinal hernia repair using routine administrative data from hospital reimbursement.

## Methods

### Routine data from the German national hospital statistics

We bought the separate data files including all principal diagnoses and operations and procedures according to the German Modification of the International Classification of Diseases—Version 10 between 2005 and 2017 from the *Statistisches Bundesamt* (German federal statistics office). Data were analysed for all principal diagnoses in the category K40 (inguinal hernia) and all procedures in the category 5-530 (inguinal hernia repair) with their respective subclassifications in both sexes. Of the latter, the subcategories 5-530.0 and 5-530.9 were counted as heriorraphies, whereas the subcategories 5-530.1, 5-530.3, and 5-530.7 were counted as hernioplasties. Recurrent hernias were assessed separately. The data were provided in separate mandatory age groups of the first year of life, 1–4, 5–9, and 10–14 years. Older children could not be included as administrative data does only separate them from adults aged 18 and 19 years in datasets since 2016. Data on children living in Germany to calculate rates per 100,000 children were also obtained from the *Statistisches Bundesamt*. The properties of these data and their pitfalls have extensively been covered elsewhere [11]. In brief, the national hospital statistics became mandatory for all hospitals in Germany in 2002 that are reimbursed via the German diagnosis related groups. All licenced, irrespective of the organising institution (public, private or church-related), hospitals have to participate and the resulting data are available from 2005 onwards [12]. Due to the mandatory nature, missing data relevant for reimbursement such as principal diagnoses, procedures, age, sex, and length of stay are almost absent as non-coded or incorrectly coded procedures will result in non-reimbursement [12]. The number of procedures and principal diagnoses is not concordant as inguinal hernia repair in children may be conducted in patients whose principal diagnosis is not the inguinal hernia, i.e. a neonate receiving inguinal hernia repair before discharge.

### Statistical analysis

Statistical analysis was conducted using R (RRID: SCR_001905) (version 3.5.3) with its generic stats4-package (version 3.5.3) unless explicitly stated otherwise [13]. Changes over time were assessed via ordinary least squares linear regression if its assumptions were fulfilled, as performed before on similar data [14–16]. We checked normality of residuals with the Kolmogorow-Smirnow-test, homoscedasticity with the *F*-Test—both supported by visual analysis of QQ-plots [17]—and searched for outliers with the Bonferroni outlier test supported by the use of Cook’s distance using the olsrr-package (version 0.5.3) [18]. If outliers or heteroscedasticity were present, we used robust regression with the weighted MM-estimator and heteroscedasticity was accounted by heteroscedasticity and autocorrelation corrected standard error provided by the *lmrob*-function in the robustbase-package (version 0.93-6) [19]. Robust regression graphs were fitted with the ggplot2-package (version 3.3) via the *rlm*-method from the MASS-package (version 7.3-51-5) [20]. A normal distribution of residuals for between-groups comparison was also assessed by the Kolmogorow-Smirnow-test and visual inspection of QQ-plots. The omnibus-test for the age groups was the van der Waerden-test followed by the Conover-Iman-test posthoc from the PMCMR-package (version 4.3) [21] based on the recommendations derived from simulation studies [22] as done before [23]. Diagnoses and procedures were compared between sexes with the Mann–Whitney-Wilcoxon *U*-test. As regular confidence intervals require a normal distribution of data, bias-corrected accelerated bootstrap confidence intervals with 10,000 repetitions [24, 25] have been calculated using the *smean.cl.boot*-function from the Hmisc-package (version 4.4-0) [26, 27] for point estimates and for mean differences by bootstrapping the mean differences using the *two.boot*-function from the simpleboot-package (version 1.1-7) [28] to supply it to the *boot.ci*-function from the boot-package (version 1.3-24) [29]. We accounted for multiple testing by using the Benjamini-Hochberg-procedure [30, 31].

## Results

We included 121,928 diagnoses of inguinal hernias between 2005 and 2017, of which 97,057 occurred in males and 24,871 in females. 105,826 hernia diagnoses were unilateral, of them, 84,975 were present in males and 20,851 in females, whereas 16,102 were bilateral hernias, among them 12,082 occurred in males and 4020 in females. Incarcerated hernias were present in 19,629 cases, 18,401 were unilateral and 1228 bilateral, but inguinal hernias with already gangrenous bowel were a rarity with only 388 cases. Of the hernia repairs, 162,748 were conducted via the open approach, of which 125,975 hernias were treated by herniorrhaphy and 36,381 by hernioplasty, and only 2985 cases were treated by the laparoscopic approach.

**Fig. 1:**
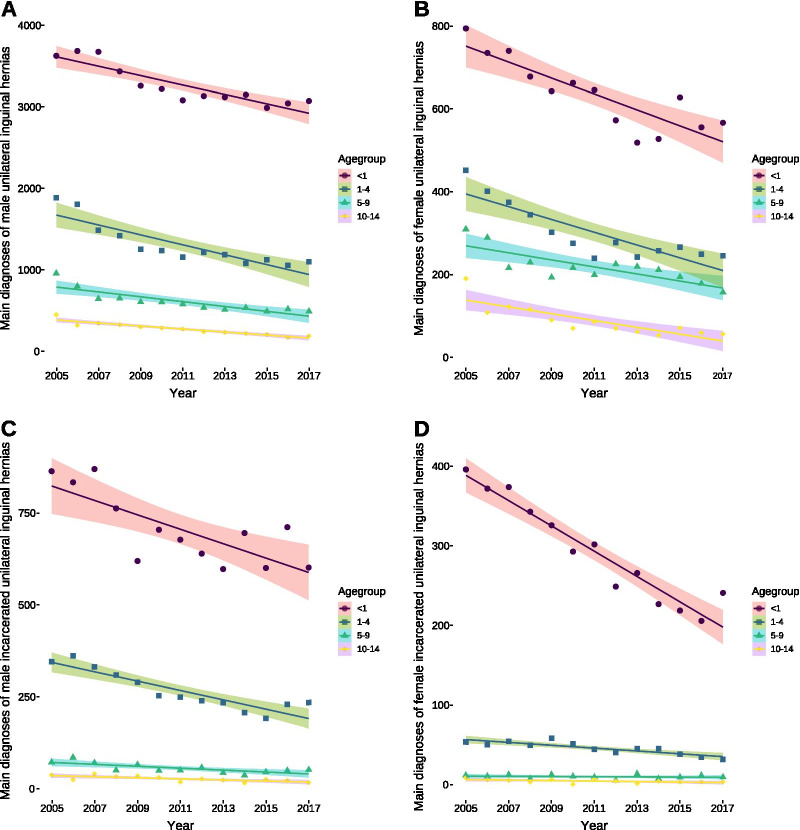
Patients diagnosed with unilateral inguinal hernias over time. **A** Decreasing numbers of male unilateral inguinal hernias per year in all age groups [< 1: 58 (95% confidence interval: 39–77, *P* < 0.0001); 1–4: 61 (95% confidence interval: 39–83, *P* < 0.0001); 5–9: 30 (95% confidence interval: 18–41, *P* = 0.0002); 10–14: 19 (95% confidence interval: 14–23, *P* < 0.0001)]. **B** Decreasing numbers of female unilateral hernias per year in all age groups [< 1: 19 (95% confidence interval: 12–26, *P* = 0.0001); 1–4: 15 (95% confidence interval: 10–21, *P* = 0.0001); 5–9: 8 (95% confidence interval: 3–14, *P* = 0.005); 10–14: 8 (95% confidence interval: 5–12, *P* = 0.0003)]. **C** Decreasing numbers of male unilateral incarcerated inguinal hernias per year in all age groups [< 1: 20 (95% confidence interval: 9–30, *P* = 0.0002); 1–4: 13 (95% confidence interval: 9–17, *P* < 0.0001); 5–9: 3 (95% confidence interval: 1–4, *P* = 0.003); 10–14: 1.5 (95% confidence interval: 0.6–2.4, *P* = 0.0043)]. **D** Decreasing numbers of female unilateral incarcerated inguinal hernias per year in the first year of life [16 (95% confidence interval: 13–19, *P* < 0.0001)] and until the age of 4 years [2 (95% confidence interval: 1–3; *P* = 0.0001)], whereas the numbers remained unchanged in older patients [5–9: − 0.1 (95% confidence interval: − 0.5–0.3, *P* = 0.501); 10–14: − 0.3 (95% confidence interval: − 0.6–0.03, *P* = 0.0665)]. All data were analyses with ordinary least squares regression except for female unilateral hernias in the age group between five and nine years

**Fig. 2:**
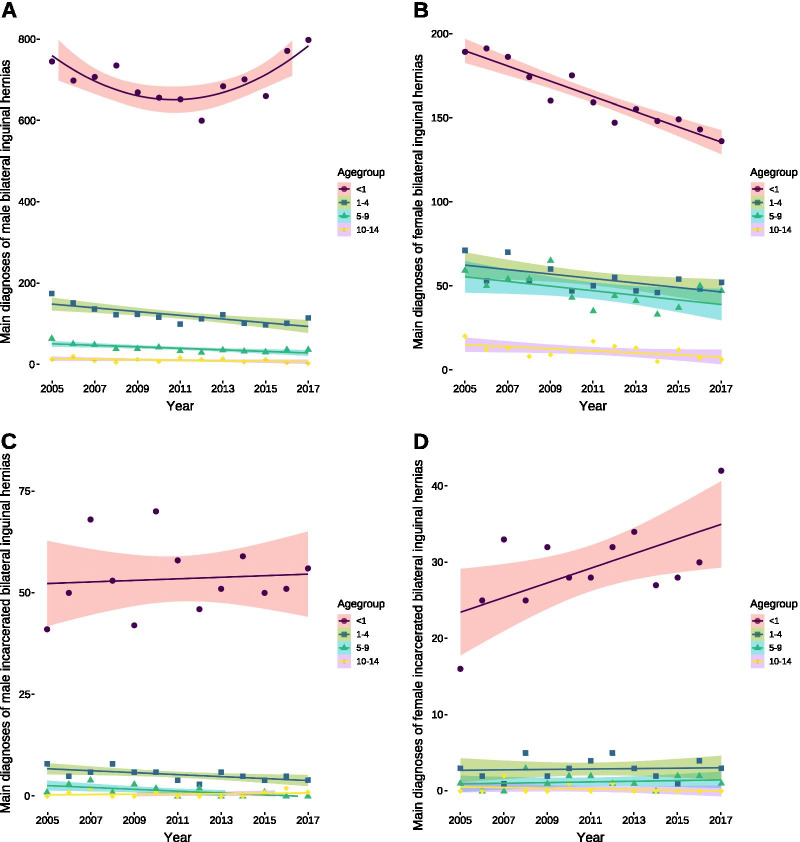
Patients diagnosed with bilateral inguinal hernias. **A** Quadratic relationship for male bilateral hernias per year in the first year of life [12 (95% confidence interval: 9–15, *P* = 0.002)] and decreasing numbers of patients in the age groups between one and four [5 (95% confidence interval: 2–7, *P* = 0.001)] as well as between 5 and 9 years [2 (95% confidence interval: 1–3, *P* = 0.0023)] over time, whereas there was no difference in the age group between 10 and 14 years [0.6 (95% confidence interval: 0.1–1.4, *P* = 0.0957)]. **B** Decreasing numbers of female bilateral inguinal hernias per year in the first year of life [5 (95% confidence interval: 4–6, *P* < 0.0001)] and in patients aged between 5 and 9 years [1.4 (95% confidence interval: 0.03–2.7, *P* = 0.0453)]. The numbers did not change for the other two age groups [1–4: 1.3 (95% confidence interval: − 3–0.3, *P* = 0.1041); 10–14: 0.6 (95% confidence interval: − 1.2–0.007, *P* = 0.0521)]. **C** Decreasing numbers of male bilateral incarcerated hernias per year in the age groups between one and four [0.2 (95% confidence interval: 0.04–0.4, *P* = 0.0219)] and also in that between 5 and 9 years of age [0.2 (95% confidence interval: 0.04–0.4, *P* = 0.0228)], whereas the number did not change for the two other age groups [< 1: 0.2 (95% confidence interval: − 1.3–1.7, *P* = 0.781); 10–14: 0.03 (95% confidence interval: − 0.1–0.2, *P* = 0.585)]. **D** Increasing numbers of female bilateral incarcerated inguinal hernias per year in the first year of life [1 (95% confidence interval: 0.2–1.8, *P* = 0.0235)], whereas the numbers remained similar in the other age groups [1–4: 0.03 (95% confidence interval: − 0.2–0.3, *P* = 0.792; 5–9: 0.05 (95% confidence interval: − 0.1–0.2, *P* = 0.546); 10–14: − 0.05 (95% confidence interval: − 0.15–0.06, *P* = 0.369)]. All data was analysed using ordinary least squares regression except for bilateral incarcerated inguinal hernias in males aged between five and nine years as well as for female bilateral inguinal hernias in the age group between one and four years

We found decreasing numbers of unilateral inguinal hernias in both males (Fig. 1A) and females (Fig. 1B) in all groups based on the numbers of principal diagnoses between 2005 and 2017. Incarcerated unilateral inguinal hernias were also shrinking over time in males in all age groups (Fig. 1C) and females up to 4 years of age, whereas they remained unchanged for older girls (Fig. 1D). Likewise, the number of male bilateral inguinal hernias decreased in the age groups between 1–4 and 5–9 years, remained unchanged in those up to 14 years of age, but decreased in males in their first year of life until 2012 and followed by a subsequent rise similar to the increasing number of live births since 2011 (Fig. 2A). This picture was slightly different for female bilateral hernias that decreased for those in their first year of life and those between 5 and 9 years, whereas it remained unchanged for girls aged 1–4 years and those between 10 and 14 years of age (Fig. 2B). The analysis for male bilateral incarcerated hernias found a decrease in the age groups between 1 and 4 years and between 5 and 9 years, but the change was almost non-existent with a reduction of 0.2 cases (95% confidence interval: 0.04–0.4) per year, whereas the numbers did not change for the remaining age groups (Fig. 2C). On the contrary, the number of bilateral incarcerated female inguinal hernias increased by one case per year (95% confidence interval: 0.2–1.8) and remained unchanged for the other age groups (Fig. 2D). There was a clear pattern for the frequency of hernias among the age groups: The younger the children were, the higher the number of diagnosed inguinal hernias was, with the exception of similar numbers for female bilateral inguinal hernias between the ages of 1–4 years and 5–9 years [1–4: 54 (95% confidence interval: 51–59) *vs.* 5–9: 47 (95% confidence interval: 42–52), *P* = 0.082] [Table 1]. Likeswise, there was no difference in incarcerated bilateral hernias in males between the age groups of 5–9 years and those up to 14 years of age [5–9: 1.3 (95% confidence interval: 0.6–2.1) *vs.* 0.6 (95% confidence interval: 0.2–1), *P* = 0.17] [Table 1].

**Table 1:** Types of hernia according to recorded main diagnoses compared by age group separately by sexes

Type of hernia	Sex	Age group	Mean (95% CI)	Omnibus test	Groupwise comparison
Unilateral hernia	M	< 1	3264 (3140–3403)	Χ^2^ = 45.192 df = 3 *P* < 0.0001	For all comparisons *P* < 0.0001
		1–4	1307 (1176–1453)		
		5–9	609 (546–689)		
		10–14	273 (72–315)		
	F	< 1	635 (593–681)	Χ^2^ = 43.899 df = 3 *P* < 0.0001	1–4 *vs.* 5–9: *P* = 0.0007 Other comparisons *P* < 0.0001
		1–4	302 (271–341)		
		5–9	218 (199–241)		
		10–14	89 (72–113)		
Incarcerated unilateral hernia	M	< 1	706 (659–760)	Χ^2^ = 45.021 df = 3 *P* < 0.0001	For all comparisons P < 0.0001
		1–4	268 (241–297)		
		5–9	57 (51–65)		
		10–14	28 (25–32)		
	F	< 1	293 (261–326)	Χ^2^ = 44.421 df = 3 *P* < 0.0001	For all comparisons *P* < 0.0001
		1–4	47 (42–51)		
		5–9	11 (9–12)		
		10–14	5 (4–6)		
Bilateral hernia	M	< 1	697 (670–725)	Χ^2^ = 45.216 df = 3 *P* < 0.0001	For all comparisons *P* < 0.0001
		1–4	121 (110–133)		
		5–9	39 (35–44)		
		10–14	11 (8–13)		
	F	< 1	163 (153–173)	Χ^2^ = 42.799 df = 3 *P* < 0.0001	1–4 *vs.* 5–9: *P* = 0.082 Other comparisons *P* < 0.0001
		1–4	54 (51–59)		
		5–9	47 (42–52)		
		10–14	11 (9–14)		
Incarcerated bilateral hernia	M	< 1	54 (49–58)	Χ^2^ = 41.787 df = 3 *P* < 0.0001	5–9 *vs.* 10–14: *P* = 0.17 Other comparisons *P* < 0.0001
		1–4	5.4 (4.6–6.2)		
		5–9	1.3 (0.6–2.1)		
		10–14	0.6 (0.2–1)		
	F	< 1	29 (26–32)	Χ^2^ = 40.94 df = 3 *P* < 0.0001	1–4 *vs.* 5–9: *P* = 0.0006 5–9 *vs.* 10–14: *P* = 0.0017 Other comparisons *P* < 0.0001
		1–4	3 (2–4)		
		5–9	1.2 (0.6–1.8)		
		10–14	0.3 (0–0.7)		

Confidence intervals were obtained via bias-corrected, accelerated bootstrap with 10,000 repetitions. The different age groups among the types of hernias were compared by van der Waerden’s test as the omnibus-test followed by groupwise comparisons via the Conover-Iman test with correction for multiple comparisons using the method of Benjamini-Hochberg

*M* male, *F* female, *CI* confidence interval

**Fig. 3:**
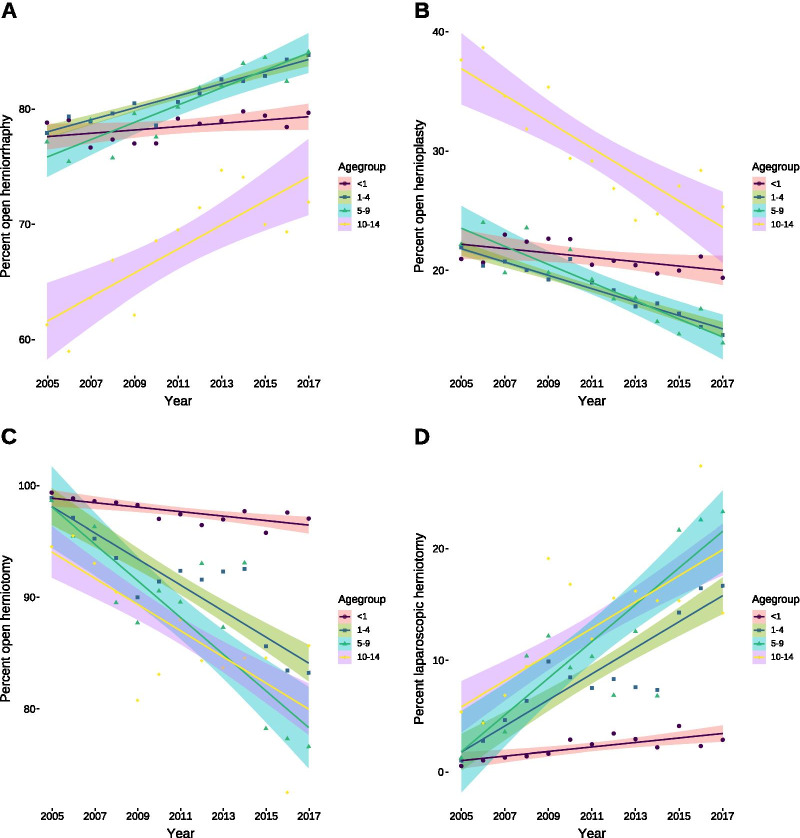
Relevant changes in operative procedures for inguinal hernias over time. **A** Increasing percentages of open herniorrhaphys per year in males in all age groups [1–4: 0.5% (95% confidence interval: 0.4–0.7, *P* < 0.0001); 5–9: 0.8% (95% confidence interval: 0.5–1, *P* < 0.0001); 10–14: 1.1% (95% confidence interval: 0.6–1.5, *P* = 0.0006)] except the first year of life [0.14% (95% confidence interval: − 0.04–0.34, *P* = 0.11]. **B** Concomitant yearly decrease in percentages of open hernioplastys in males in all age groups [1–4: 0.6% (95% confidence interval: 0.4–0.7, *P* < 0.0001); 5–9: 0.8% (95% confidence interval: 0.5–1, *P* < 0.0001); 10–14: 1.1% (95% confidence interval: 0.7–1.5, *P* < 0.0001)] excluding the first year of life [0.2% (95% confidence interval: 0.06–0.4, *P* = 0.1158)]. **C** Decreasing per year percentages of open herniotomys in females in all age groups [< 1: 0.2% (95% confidence interval: 0.08–0.3, *P* = 0.0045); 1–4: 1.1% (95% confidence interval: 0.7–1.5, *P* < 0.0001); 5–9: 1.6% (95% confidence interval: 0.9–2.2, *P* = 0.0004); 10–14: 1.2% (95% confidence interval: 0.4–1.9, *P* = 0.0046)]. **D** Concomitant yearly increase in the percentage of laparoscopic herniotomys in females of all age groups [< 1: 0.2% (95% confidence interval: 0.08–0.3, *P* = 0.0046); 1–4: 1.1% (95% confidence interval: 0.7–1.5; *P* < 0.0001); 5–9: 1.6% (95% confidence interval: 0.9–2.2, *P* = 0.0004); 10–14: 1.2% (95% confidence interval: 0.4–1.9, *P* = 0.0046)]. All analyses were conducted using ordinary least square regression except for open herniorrhaphy/hernioplasty in males in the first year of life and open/laparoscopic herniotomys in females of the same age group

The investigation of procedures often showed no or only marginal differences between the age groups (Table 2), although with some exceptions: In males, the distribution among simple herniorrhaphy and hernioplasty was shifted towards herniorrhaphy, particularly in the age group of 10 years and above, but remained unchanged in the first year of life (Fig. 3A, B). In females, the percentage of laparoscopically conducted hernia repairs increased, only slightly in the first year of life, but more than 1% per year in the remaining age groups (Fig. 3C) with a concomitant decrease in open repairs (Fig. 3D). In addition, open hernioplasty was conducted with an increased frequency of more than 10% in males and almost 20% in females, compared to younger age groups (Table 2). Comparison between males and females revealed that open repair was predominantly conducted in males, whereas laparoscopy was more often used in females (Table 3). Likewise, herniorrhaphy was more prevalent in males, whereas hernioplasty was in females (Table 3). The percentage of recurrent hernia repairs was slightly higher in males with the exception of the age group between 10 and 14 years of age, in which there were no difference (Δ = 0.7% (95% confidence interval: − 0.5–1.4), *P* = 0.137). The distribution of the procedures among the recurrent hernitomies could not be suitably assessed due to the rarity of events; an aspect that is also true for the use of alloplastic materials in primary hernia repair as well as bowel resection without concomitant laparotomy. Qualitatively, recurrent hernia repair was largely a domain of open surgery with hernioplasty, whereas the use of alloplastic materials was—as expected—a rarity. Laparoscopic herniotomy was a domain of transabdominal repair, whereas extraperitoneal repair was rarely used.

**Table 2:** Percentages of hernia repair procedures according to recorded procedures in total compared by age group separately by sexes

Type of procedure	Sex	Age group	Mean (95% CI)	Omnibus test	Groupwise comparison		
Open hernia repair	M	< 1	98.8% (98.4–99.1)	Χ^2^ = 20.508 df = 3 *P* = 0.0001	< 1 *vs.* 1–4: *P* = 0.5781	< 1 *vs.* 5–9: *P* = 0.0727	
		1–4	98.8% (98.4–99.2)		< 1 *vs.* 10–14: *P* = 0.0001	1–4 *vs.* 5–9: *P* = 0.025	
		5–9	98.1% (97.4–98.7)		1–4 *vs.* 10–14: *P* < 0.0001		
		10–14	97% (96.2–97.6)		5–9 *vs.* 10–14: *P* = 0.0214		
	F	< 1	97.7% (97.2–98.2)	Χ^2^ = 23.199 df = 3 *P* < 0.0001	< 1 *vs.* 1–4: *P* = 0.001		< 1 *vs.*: 5–9: *P* < 0.0001
		1–4	91.4% (88.8–93.8)		< 1 *vs.* 10–14: *P* < 0.0001		1–4 *vs.* 5–9: *P* = 0.288
		5–9	88.8% (85–92.4)		1–4 *vs.* 10–14: *P* = 0.045		
		10–14	86.3% (82.9–89.4)		5–9 *vs.* 10–14: *P* = 0.288		
Laparoscopic hernia repair	M	< 1	1.2% (0.9–1.6)	Χ^2^ = 21.114 df = 3 P < 0.0001	< 1 *vs.* 1–4: *P* = 0.589		< 1 *vs.*: 5–9: *P* = 0.074
		1–4	1.2% (0.8–1.6)		< 1 *vs.* 10–14: *P* < 0.0001		1–4 *vs.* 5–9: *P* = 0.027
		5–9	1.9% (1.3–2.6)		1–4 *vs.* 10–14: *P* < 0.0001		
		10–14	2.9% (2.4–3.6)		5–9 *vs.* 10–14: *P* = 0.014		
	F	< 1	2.3% (1.8–2.8)	Χ^2^ = 23.199 df = 3 *P* < 0.0001	< 1 *vs.* 1–4: *P* = 0.001		< 1 *vs.*: 5–9: *P* < 0.0001
		1–4	8.6% (6.2–11.2)		< 1 *vs.* 10–14: *P* < 0.0001		1–4 *vs.* 5–9: *P* = 0.288
		5–9	11.2% (7.6–15)		1–4 *vs.* 10–14: *P* = 0.045		
		10–14	13.7% (10.6–17.1)		5–9 *vs.* 10–14: *P* = 0.288		
Open herniorraphy	M	< 1	78.5% (77.9–79)	Χ^2^ = 32.247 df = 3 *P* < 0.0001	< 1 *vs.* 1–4: *P* = 0.0051		
		1–4	81% (80–82.2)		< 1 *vs.* 5–9: *P* = 0.0254		
		5–9	80.3% (78.7–82)		1–4 *vs.* 5–9: 0.4867		
		10–14	67.9% (65–70.4)		Other comparisons: *P* < 0.0001		
	F	< 1	69.9% (69–70.9)	Χ^2^ = 28.269 df = 3 *P* < 0.0001	< 1 *vs.* 1–4: *P* = 0.84		
		1–4	69.8% (68.7–70.9)		< 1 *vs.* 5–9: *P* = 0.84		
		5–9	69.6% (67.8–71.3)		1–4 *vs.* 5–9: *P* = 0.84		
		10–14	50.4% (47.2–53.8)		Other comparisons: *P* < 0.0001		
Open hernioplasty	M	< 1	21.1% (20.5–21.7)	Χ^2^ = 32.246 df = 3 *P* < 0.0001	< 1 *vs.* 1–4: *P* = 0.0071		
		1–4	18.6% (17.3–19.7)		< 1 *vs.* 5–9: *P* = 0.0147		
		5–9	19% (17.3–20.7)		1–4 *vs.* 5–9: 0.7215		
		10–14	30.2% (27.8–33)		Other comparisons: *P* < 0.0001		
	F	< 1	29.9% (28.8–30.8)	Χ^2^ = 27.765 df = 3 *P* < 0.0001	< 1 *vs.* 1–4: *P* = 0.79		
		1–4	30.1% (28.8–31.1)		< 1 *vs.* 5–9: *P* = 0.79		
		5–9	29.7% (27.8–31.7)		1–4 *vs.* 5–9: 0.74		
		10–14	48% (43.7–52)		Other comparisons: *P* < 0.0001		
Recurrent hernia	M	< 1	0.6% (0.5–0.7)	Χ^2^ = 24.698 df = 3 *P* < 0.0001	< 1 *vs.* 1–4: *P* = 0.022		< 1 *vs.* 5–9: *P* < 0.0001
		1–4	1.6% (1.3–1.9)		< 1 *vs.* 10–14: *P* < 0.0001		1–4 vs. 5–9: P = 0.022
		5–9	2.5% (2–3)		1–4 vs. 10–14: P = 0.001		
		10–14	3.1% (2.4–3.7)		5–9 vs. 10–14: P = 0.225		
	F	< 1	0.3% (0.2–0.4)	Χ^2^ = 37.525 df = 3 *P* < 0.0001	1–4 *vs.* 5–9: *P* = 0.0865		
		1–4	1% (0.8–1.2)		5–9 *vs.* 10–14: *P* = 0.0001		
		5–9	1.3% (1.1–1.6)		Other comparisons: *P* < 0.0001		
		10–14	2.6% (2–3.2)				

Confidence intervals were obtained via bias-corrected, accelerated bootstrap with 10,000 repetitions. The different age groups among the types of hernias were compared by van der Waerden’s test as the omnibus-test followed by groupwise comparisons via the Conover-Iman test with correction for multiple comparisons using the method of Benjamini-Hochberg

*M* male, *F* female, *CI* confidence interval

**Table 3:** Comparison of shares of procedures between males and females by age group

Type of procedure	Age group	Difference of means (95% CI)	Mann–Whitney estimate	*P*-value
Open hernia repair	< 1	1.1% (0.4–1.7)	U = 0.1775	0.0056
	1–4	7.4% (5.2–10.3)	U = 0.0355	< 0.0001
	5–9	9.3% (5.9–13.7)	U = 0.0592	0.0002
	10–14	10.7% (7.6–14.2)	U = 0.0178	< 0.0001
Laparoscopic hernia repair	< 1	− 1.1% (− 1.7 to − 0.4)	U = 0.8225	0.0056
	1–4	− 7.4% (− 10.2 to − 5.1)	U = 0.9645	< 0.0001
	5–9	− 9.3% (− 13.7 to − 5.9)	U = 0.9408	0.0002
	10–14	− 10.7% (− 14.4 to − 7.8)	U = 0.9823	< 0.0001
Open herniorrhaphy	< 1	8.6% (7.2–9.5)	U = 0	< 0.0001
	1–4	11.2% (9.6–12.8)	U = 0	< 0.0001
	5–9	10.7% (8.3–13.2)	U = 0	< 0.0001
	10–14	17.5% (12.9–21.4)	U = 0.0178	< 0.0001
Open hernioplasty	< 1	− 8.8% (− 9.8 to − 7.5)	U = 1	< 0.0001
	1–4	− 11.5% (− 13.3 to − 9.7)	U = 1	< 0.0001
	5–9	− 10.7% (− 13.4 to − 8.2)	U = 1	< 0.0001
	10–14	− 17.8% (− 22.2 to − 12.3)	U = 0.9645	< 0.0001
Recurrent hernia	< 1	0.3% (0.2–0.4)	U = 0.1065	0.0007
	1–4	0.6% (0.2–0.9)	U = 0.1124	0.0009
	5–9	1.2% (0.5–1.8)	U = 0.1183	0.001
	10–14	0.7% (− 0.5–1.4)	U = 0.3254	0.137

Confidence intervals were obtained via bias-corrected, accelerated bootstrap with 10,000 repetitions. Comparisons were undertaken via Mann–Whitney-Wilcoxon *U*-test with correction for multiple comparisons according to Benjamini-Hochberg

*CI* confidence interval

**Fig. 4:**
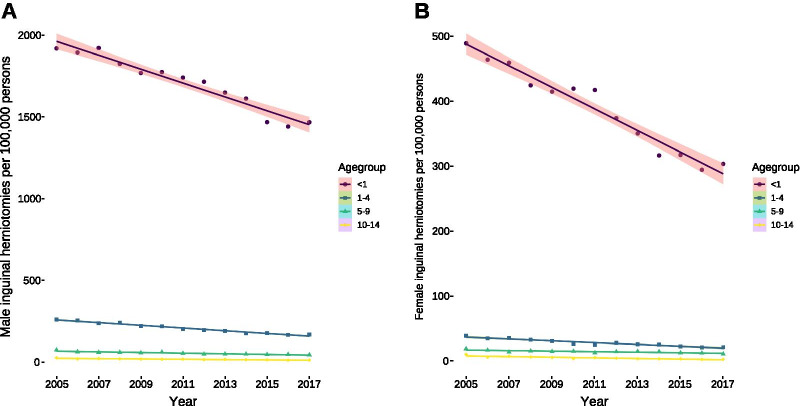
Rates of inguinal herniotomies per 100,000 persons per year. **A** Rates of inguinal herniotomies per 100,000 males per year decreased for all age groups [< 1: 42 (95% confidence interval: 49–36, *P* < 0.0001); 1–4: 8 (95% confidence interval: 9–7, *P* < 0.0001); 5–9: 2 (95% confidence interval: 2.5–1.5, *P* < 0.0001); 10–14: 1 (95% confidence interval: 1.4–0.5; *P* = 0.0005)]. **B** Rates of inguinal herniotomies per 100,000 females per year decreased for all age groups [< 1: 17 (95% confidence interval: 19–14, *P* < 0.0001); 1–4: 1.4 (95% confidence interval: 1.7–1.1, *P* < 0.0001); 5–9: 0.4 (95% confidence interval: 0.6–0.2, *P* < 0.0006); 10–14: 0.4 (95% confidence interval: 0.5–0.3, *P* < 0.0001)]. All analyses were conducted using ordinary least square regression except for the age group between 10 and 14 years of age of both sexes

To adequately assess and compare the development of inguinal herniotomies between the different years—the number of children were declining in Germany with a sole in 2011 and increasing afterwards—we calculated the rates of herniotomies in inpatients per 100,000 children. They were decreasing over time in all age groups, in both males (Fig. 4A) and females (Fig. 4B), despite the increase of children in the respective age groups since 2011. The comparison between both sexes revealed that there was a substantial difference particularly in the first year of life with 1319 herniotomies (95% confidence interval: 1215–11,407, *U* = 0, *P* < 0.0001) more in boys than girls. This difference was much less pronounced in the age group between 1 and 4 years (Δ = 182, 95% confidence interval: 166–200, *U* = 0, *P* < 0.0001), 5–9 years (Δ = 44, 95% confidence interval: 40–49, *U* = 0, *P* < 0.0001), and 10–14 years of age (Δ = 16, 95% confidence interval: 14–18, *U* = 0, *P* < 0.0001).

In order to assess the differences between the data on principal diagnoses and the data on operations and procedures, we assessed the difference between principal diagnoses and procedures by adding all principal diagnoses and in case of bilateral hernias by multiplying their number with 2 and subtracted the number of procedures from this result. This revealed that excess procedures in male inpatients in every age group [< 1: 755 (95% confidence interval: 699–812); 1–4: 1316 (95% confidence interval: 1236–1398); 5–9: 412 (95% confidence interval: 375–452); 10–14: 136 (95% confidence interval: 126–147)], which demonstrates that up to almost 50% of inguinal herniotomies in the age group between 1 and 4 years were conducted when a diagnosis other than inguinal hernia was the main diagnosis during the hospital stay. This difference is less pronounced in females, in which excess procedures did occur in the first year of life ($\overline{x}$ = 50, 95% confidence interval: 30–75) and in the age group between 10 and 14 years ($\overline{x}$ = 13, 95% confidence interval: 8–18), but not in the remaining age groups.

**Fig. 5:**
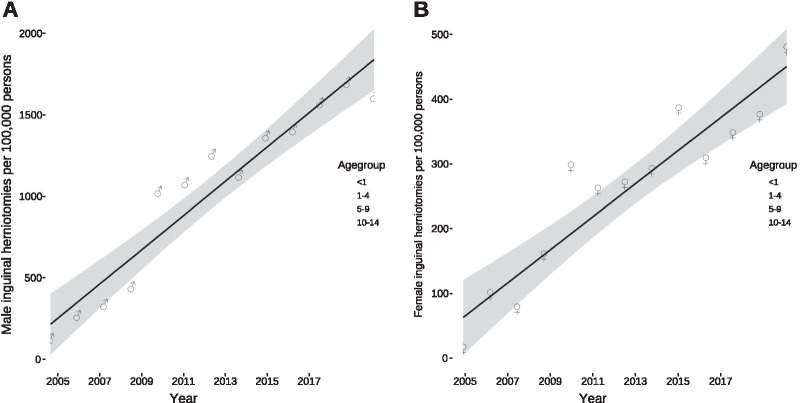
Inguinal hernia repairs performed by paediatric surgeons. **A** Percentages of inguinal hernia repair in males conducted in paediatric surgical departments increased by 1.6% (95% confidence interval: 1.3–1.9, *P* < 0.0001) per year. **B** Percentages of inguinal hernia repairs in females by paediatric surgeons increased by 1.5% (95% confidence interval: 1.1–1.8, *P* < 0.0001). Both analyses were conducted using ordinary least square regression

We finally assessed the proportion of diagnosed hernias that were treated by paediatric surgeons and found that their share of inpatients increased every year for both males (Fig. 5A) and females (Fig. 5B).

## Discussion

In contrast to inguinal hernia surgery in adults [8, 9], routine administrative data have been seldom used for paediatric inguinal hernia surgery. The ratio of male and female inguinal hernias was concordant with previous reports: There numbers of inguinal hernias in males were almost four times higher than in females whose hernias were more often incarcerated than those in boys [3]. Our results also were comparable to other reports describing the age distribution among affected children: The younger the patient, the higher the probability to be diagnosed with an inguinal hernia irrespective of sex or ethnicity [2–4]. A reduction of inguinal hernia repairs over time has been reported in the United States [32], in Korea [33], and in England with a substantial reduction of more than 60% in the first year of life and up to almost 90% until the age of 14 years without a concomitant increase in incarcerated hernias [4]. The reason for these substantial changes remained unclear to the authors, but others were encouraged to assess their data for a similar development [4], which we may now report for Germany, too. Similar to others [4, 32], we are unable to explain this decline in the number of inguinal hernia repairs. In order to separate the children identified by the main diagnosis, we also calculated rates of inguinal herniotomies per 100,000 children and also found they were decreasing during the study period. Therefore, we may exclude that this decline is caused by inguinal hernia repairs conducted in parallel to other operations such as orchidopexy, which would explain high rates of funicolysis during inguinal hernia repair in the age group between 1 and 4 years of age, in which the majority of orchidopexies is conducted in Germany [34]. As the number of incarcerated inguinal hernias is also decreasing, it is unlikely that there is a shift towards incarcerated hernias. These might have been expected, because children—unlike adults in which many incarcerated hernias are not on the waiting list for elective surgery[35]—have an increased risk of incarceration with waiting time until elective repair [36, 37].

The reduction in numbers from hernioplasty towards herniorrhaphy in the study period may be closely linked to the shift of operations performed by paediatric surgeons, because a survey among general and paediatric surgeons showed that general surgeons were much more likely than paediatric surgeons to choose an adult-type hernia repair with either muscle hernioplasty or mesh-implantation [38]. This suggests a tendency to stick with the approach that one is proficient with, because there is a relevant learning curve to learn the open [39] or the transabdominal laparoscopic approach [40]. Taking into account the steadily increasing number of paediatric surgeons in Germany [41] would support the notion that their expertise in treating children becomes more broadly available and thus supplant adult surgeons using their approach of hernioplasty.

Laparoscopic inguinal hernia repair was—unlike in adults [9]—rare in children and was only increasingly used in females older than 1 year of age. While earlier, smaller, comparisons of the open and laparoscopic approaches using unmatched data reported favourable outcomes for the laparoscopic procedure [42], a recent study employed propensity-matching and described several downsides of the laparoscopic approach such as always longer anaesthesia times for both uni- and bilateral inguinal hernia repairs and much higher recurrence rate compared to the open approach [43]. As propensity-matching is thought to increase the precision of a comparison, it might result in more credible data compared to crude comparisons.

It may theoretically be the case that laparoscopic procedures are more often conducted as ambulatory surgery and thus not appear in the inpatient procedure records. However, even the most recent technical descriptions of laparoscopic hernia surgery describe their use on inpatients, which do not support this notion. In addition, there is the problem of missing cost-effectiveness of hernia surgery per se [44], but in particular for ambulatory endoscopic surgery in which reimbursements do not cover the procedural costs [45]. It has thus been feared, based on a survey in Switzerland, which has a medical system broadly comparable to Germany, that upcoding may become an issue in using inpatient administrative data due to the resulting distortion [46]. However, a relevant shift of operations towards office-based surgeons is unlikely as the negative reimbursement differences are rather large in inguinal hernia surgery compared to other surgeries that could potentially be conducted office-based [47]. One would also have expected an increase in double-sided inguinal hernia repairs as they can be identified more often when using laparoscopy [48], but the incidence of double-sided hernia diagnoses was decreasing, too. Taking into account that a survey among European paediatric surgeons found open paediatric inguinal hernia repair to be the procedure of choice for more than 80% of respondents [49], which mirrors the 85% of inguinal hernia repairs via the open approach conducted in children’s hospitals in the United States [10]. In Denmark, inguinal hernia repair in children is almost exclusively [50] and in Korea to a large proportion [33] the domain of open surgery, our finding in Germany might just reflect the clinical reality in German inpatient paediatric surgery, in which the open approach was long considered the “gold standard” [51].

Using administrative data from the national reimbursement statistics has several limitations: It only covers inpatients, whereas all office-based procedures are not included, which might distort the numbers, particularly in older age groups, which are increasingly treated office-based compared to their infant counterparts. However, the reimbursement of an office-based hernia repair is at around 50% of the inpatient case [52]. Consequently, the relative share of office-based inguinal hernia surgery is around 15% of all inguinal herniotomies conducted in Germany, which is in sharp contrast to the rest of the world, but caused by the incentive of the much higher inpatient reimbursement [53]. As a result, the small share of office-based procedures missed by our data is unlikely to have a relevant effect to our results. Another limitation of our data is its case-based approach, which could result in the same patient being having two cases in the same year, for example if he would be transferred from one to another hospital or in case of a metachronous hernia on the other side. These limitations might partially be overcome by data from the statutory health insurances, which are patient- instead of case-based and also includes data from procedures conducted on outpatients in hospitals [16, 17].

## Conclusion

Paediatric inguinal hernia surgery in inpatient children is largely conducted on younger children with a predominance of boys with rates similar to other countries. The treatment of choice is open surgery with the exception of females after the first year of life and increasingly performed by paediatric surgeons. Since 2005, almost all hernia types became less frequent every year; a development that has been observed in other countries before, too, but an explanation for this development is missing.

## Data Availability

The data that support the findings of this study are available from the *Statistisches Bundesamt* (German federal statistics office) but restrictions apply to the availability of these data, which were used under license for the current study, and so are not publicly available. Data are however available from the authors upon reasonable request and with permission of the *Statistisches Bundesamt* (German federal statistics office).
